# The role of the ALKBH5 RNA demethylase in invasive breast cancer

**DOI:** 10.1007/s12672-024-01205-8

**Published:** 2024-08-11

**Authors:** Corinne L. Woodcock, Mansour Alsaleem, Michael S. Toss, Jennifer Lothion-Roy, Anna E. Harris, Jennie N. Jeyapalan, Nataliya Blatt, Albert A. Rizvanov, Regina R. Miftakhova, Yousif A. Kariri, Srinivasan Madhusudan, Andrew R. Green, Catrin S. Rutland, Rupert G. Fray, Emad A. Rakha, Nigel P. Mongan

**Affiliations:** 1 University of Nottingham Biodiscovery Institute, University of Nottingham, Nottingham, UK; 2 Faculty of Medicine and Health Science, School of Veterinary Medicine and Science, University of Nottingham, Nottingham, UK; 3 Nottingham Breast Cancer Research Centre, School of Medicine, Academic Unit for Translational Medical Sciences, University of Nottingham, Nottingham, UK; 4 Unit of Scientific Research, Applied College, Qassim University, Qassim, Saudi Arabia; 5 Institute for Fundamental Medicine and Science, Kazan Federal University, Kazan, Tatarstan Russia; 6 Department of Clinical Laboratory Science, Faculty of Applied Medical Science, Shaqra University 33, 11961 Shaqra, Saudi Arabia; 7 Department of Histopathology, Nottingham University Hospitals NHS Trust, Nottingham City Hospital, Nottingham, UK; 8 Department of Pharmacology, Weill Cornell Medicine, New York, NY USA; 9 School of Biosciences, Plant Science Division, University of Nottingham, Nottingham, UK; 10 Pathology Department, Hamad General Hospital, Hamad Medical Corporation, Doha, Qatar

**Keywords:** N6-methyladenosine, Epitranscriptomics, m^6^A, Prognosis, Breast cancer

## Abstract

**Background:**

N6-methyladenosine (m^6^A) is the most common internal RNA modification and is involved in regulation of RNA and protein expression. AlkB family member 5 (ALKBH5) is a m^6^A demethylase. Given the important role of m^6^A in biological mechanisms, m^6^A and its regulators, have been implicated in many disease processes, including cancer. However, the contribution of ALKBH5 to invasive breast cancer (BC) remains poorly understood. The aim of this study was to evaluate the clinicopathological value of ALKBH5 in BC.

**Methods:**

Publicly available data were used to investigate *ALKBH5* mRNA alterations, prognostic significance, and association with clinical parameters at the genomic and transcriptomic level. Differentially expressed genes (DEGs) and enriched pathways with low or high *ALKBH5* expression were investigated. Immunohistochemistry (IHC) was used to assess ALKBH5 protein expression in a large well-characterised BC series (n = 1327) to determine the clinical significance and association of ALKBH5 expression.

**Results:**

Reduced *ALKBH5* mRNA expression was significantly associated with poor prognosis and unfavourable clinical parameters. *ALKBH5* gene harboured few mutations and/or copy number alternations, but low *ALKBH5* mRNA expression was seen. Patients with low *ALKBH5* mRNA expression had a number of differentially expressed genes and enriched pathways, including the cytokine-cytokine receptor interaction pathway. Low ALKBH5 protein expression was significantly associated with unfavourable clinical parameters associated with tumour progression including larger tumour size and worse Nottingham Prognostic Index group.

**Conclusion:**

This study implicates ALKBH5 in BC and highlights the need for further functional studies to decipher the role of ALKBH5 and RNA m^6^A methylation in BC progression.

**Supplementary Information:**

The online version contains supplementary material available at 10.1007/s12672-024-01205-8.

## Introduction

Breast cancer (BC) is the most commonly diagnosed cancer and the leading cause of cancer related mortality amongst women [1]. It is a heterogeneous group of diseases with distinct clinical, morphological, and molecular features between tumours that aid disease classification and inform treatment decision making [2].

N6-methyladenosine (m^6^A) is the most abundant internal mRNA modification and is dynamically regulated by a multiprotein complex of ‘writers’, ‘erasers’, and ‘readers’ that methylate, demethylate, and interpret the m^6^A mark, respectively [3]. The RNA methyltransferase complex is made up of methyltransferase-like 3 (METTL3), METTL14, and multiple adapter proteins [4]. AlkB family member 5 (ALKBH5) and fat-mass and obesity-associated protein (FTO) are currently the only identified m^6^A demethylases [5, 6].

The m^6^A modification is involved in a diverse set of mRNA transcription, splicing, translation, and stability functions [6–11]. Evidence is emerging that implicates the m^6^A epitranscriptomic modification in a variety of biological processes, including carcinogenesis [12–20]. Recent studies have also associated m^6^A regulators, including ALKBH5, in BC development, progression, and prognosis [21–27]. In BC, it has been reported that ALKBH5 expression is regulated by hypoxia inducible factors (HIFs), leading to increased expression of NANOG, thereby promoting the BC stem cell phenotype [28, 29]. ALKBH5 expression has also been shown to be increased in immortalised and transformed breast cell lines and tumour samples, and implicated in migration, invasion, and metastasis [22, 23, 27, 30–32]. However, the role of ALKBH5 in BC remains unclear. Therefore, this study aimed to investigate the relationship between ALKBH5 expression and clinicopathological factors in a large patient cohort and to relate this to mechanisms involving differential global gene expression identified using the TCGA-BRCA cohort of invasive BC cases stratified based on *ALKBH5* mRNA expression.

## Materials and methods

### Cell line culture conditions

Human mammary epithelial cells (HMEC), MCF10A, MCF7, T-47D, MDA-MB-453, and MDA-MB-231 breast cells were utilised. HMEC, MCF-7, T47D, MDA-MB-231, and MDA-MB-453 were generously provided by Professor Lorraine Gudas (Weill Cornell Medicine). The MCF10A were a generous gift from Dr Cinzia Allegrucci (University of Nottingham). BC cell lines MCF7, T-47D and MDA-MB-231 were grown in phenol red containing RPMI-1640 medium with L-glutamine (Gibco) supplemented with 10% foetal bovine serum (FBS) (Sigma-Aldrich), 1% penicillin–streptomycin–glutamine (Gibco), and 1 mM sodium pyruvate (Gibco). MDA-MB-453 were maintained in DMEM media (Gibco) supplemented with FBS (Sigma-Aldrich) and 1% penicillin–streptomycin–glutamine (Gibco). HMEC cells grown in HuMEC media with the addition and HuMEC supplements (Gibco). MCF10A were grown in HuMEC media with HuMEC supplements (Gibco) and 100 ng/ml cholera toxin (Sigma-Aldrich). All cells were cultured at 5% CO_2_ at 37 °C.

### Gene expression analysis

Cells were harvested for RNA using the GenElute™ Mammalian Total RNA Miniprep Kit (RTN70-1KT, Sigma-Aldrich), following manufacturer’s instructions. The qScript cDNA Synthesis Kit was used for complementary DNA (cDNA) synthesis (95047-100, Quantabio). For mRNA expression analysis, quantitative real-time polymerase chain reaction (qRT-PCR) was performed using *ALKBH5* (Hs00539502_m1) and β*-actin* (Hs01060665_g1) Taqman probes (ThermoScientific) with LightCycler^®^ 480 Probes Master (Roche Diagnostics) in a qRT-PCR machine (Bio-Rad) and the relative mRNA expression was determined by the Pfaffl method, as previously described [33].

### Western blotting

Cell lysates in final sample buffer (100 mM Tris–HCl pH 6.8, 4% SDS and 20% glycerol) were used to assess protein expression of ALKBH5 in cell lines using western blotting (n = 3). The membrane was blocked using 5% bovine serum albumin or milk for 1 h at room temperature and probed with ALKBH5 antibody overnight at 4 °C (1:5000; Novus Biologicals, NBP1-82188) or β-actin antibody (1:10,000; Invitrogen, MA515739). For secondary antibodies, goat IgG HRP anti-rabbit or goat IgG HRP anti-mouse (1:10,000; Abcam, ab6721 and ab97023) were used for 1 h at room temperature, the signal was detected using Amersham™ ECL™ Prime reagent (GE Healthcare) and image captured using a ChemiDoc™ MP Imaging System (Bio-Rad). Full uncropped western blots are displayed in Supplementary Fig. 1.

### Patient cohort

This study used a well-characterised retrospective cohort of patients diagnosed with primary invasive BC (Stage I–III, age (55, 20–87), tumour size (1.7250, 0.2–8)) at Nottingham University Hospitals NHS Trust—City Hospital Campus between 1998 and 2006 (n = 1327), as previously described [34]. The full patient demographics are described in Supplementary Table 1. This study was reviewed and approved by the Nottingham Research Ethics Committee, (approval # REC202313), and the research ethics committee of the University of Nottingham School of Veterinary Medicine and Science (approval # 2803 190814). The General Data Protection Regulation (GDPR) was applied, and informed consent obtained. The Helsinki Declaration of Human Rights was strictly observed. The Nottingham Prognostic Index (NPI) and hormone receptor status were used to inform patient management. Patients within the NPI excellent prognostic group (score ≤ 3.4) did not receive adjuvant therapy, but those patients with NPI > 3.4 received tamoxifen if ER-positive and were able to receive chemotherapy if ER-negative. Chemotherapy regimen included cyclophosphamide, methotrexate, and 5-flurouracil (CMF). Outcome data includes breast cancer specific survival (BCSS), disease free interval (DFI), and distant metastasis free survival (DMFS) [35].

### Tissue microarrays and immunohistochemical staining

The cohort was arrayed using a tissue microarray (TMA) Grand Master (3D Histech), as previously described [36]. Immunohistochemical (IHC) staining was performed on 4-μm thick TMA sections using the Novolink polymer detection system (Leica Biosystems). Heat-induced antigen epitope retrieval was performed in citrate buffer (pH 6.0) for 20 min using a microwave oven. Sections were incubated with the primary ALKBH5 antibody (1:100; Novus Biologicals, NBP1-82188) diluted in Leica antibody diluent (Leica Biosystems) at room temperature for 1 h. Slides were washed and incubated with post primary block for 30 min. Novolink polymer was applied for 30 min followed by application of 3, 3′-diaminobenzidine (DAB) chromogen for 5 min. Slides were counterstained with Novolink haematoxylin for 6 min, dehydrated, and cover slipped.

### Scoring of ALKBH5 protein expression

Stained TMA sections were scanned using a digital scanner (NanoZoomer, Hamamatsu Photonics) at × 20 magnification. High resolution images were viewed using Xplore (Phillips Pathology) to score ALKBH5 expression within the tumour cells. A modified histochemical score (H-score) was used to evaluate stained cells [37]. Staining intensity was assessed as follows: 0, negative; 1, weak; 2, moderate; 3, strong, and the percentage of the positively stained tumour cells was estimated subjectively. The final H-score was calculated by multiplying the percentage of positive cells (0–100) by the intensity (0–3), producing a total range of 0–300. Scoring was assessed independently by two researchers and an intraclass concordance of > 0.8 was confirmed.

### ALKBH5 transcriptomic data

The cBioPortal for Cancer Genomics [38, 39] was used to investigate *ALKBH5* copy number and mRNA expression alterations in BC patients utilising The Cancer Genome Atlas (TCGA) Firehose Legacy (n = 1108) [40] and the Molecular Taxonomy of Breast Cancer International Consortium (METABRIC) (n = 2509) [41, 42] cohorts. Kaplan Meier Plotter (KM-Plotter) was used to investigate the prognostic value of *ALKBH5* for overall survival (OS), relapse free survival (RFS), and distant metastasis free survival (DMFS) [43] and the Breast Cancer Gene-Expression Miner v4.7 (bc-GenExMiner v4.7) database used to investigate *ALKBH5* expression and clinical factors [44]. The UCSC Xena browser [45] was used to access the GDC TCGA BC RNA-sequencing dataset to determine *ALKBH5* expression in normal (n = 113), primary tumour (n = 1097), and metastatic samples (n = 7). Utilising the METABRIC dataset, mRNA expression in primary patients were dichotomised into low and high *ALKBH5* expression and correlated with clinical factors. Differential gene expression analysis was conducted on the primary tumour data from TCGA data set using DESeq2. Samples were dichotomised by quartile into lowest (< 4804.337) and highest (> 6911.127) expression of *ALKBH5* and significantly differentially expressed genes (DEGs) identified (fold change ± 2 and FDR-corrected p-value < 0.05). WEB-based GEne SeT AnaLysis Toolkit (WebGestalt) [46] was used to investigate over representation analysis and enrichment of KEGG pathways with the up-and down-regulated DEGs.

### Statistical analysis

Statistical analysis was performed using SPSS 24.0 statistical software (SPSS Inc.) or GraphPad Prism 8 (Dotmatics). A t-test was used to assess *ALKBH5* mRNA expression in different cell lines (n = 5/6). ALKBH5 protein expression was dichotomised into low and high expression using the X-tile software, used to identify the optimal cut-off based on the association of the protein expression and patient outcome (BCSS) [47]. This has resulted in the following, for nuclear (0–300; low expression ≤ 135, high expression > 135), cytoplasmic (0–300; low expression ≤ 90, high expression > 90), and for nuclear and cytoplasmic staining combined (0–300 calculated but the scores were added together and dived by 2; low expression ≤ 88, high expression > 88). The chi-square test (χ^2^) was performed to analyse the relationships between expression and categorical variables. Survival curves were analysed by the KM and log rank test. The p*-*values ≤ 0.05 were considered significant. Data is reported in line with the REMARK guidance [48].

## Results

**Fig. 1:**
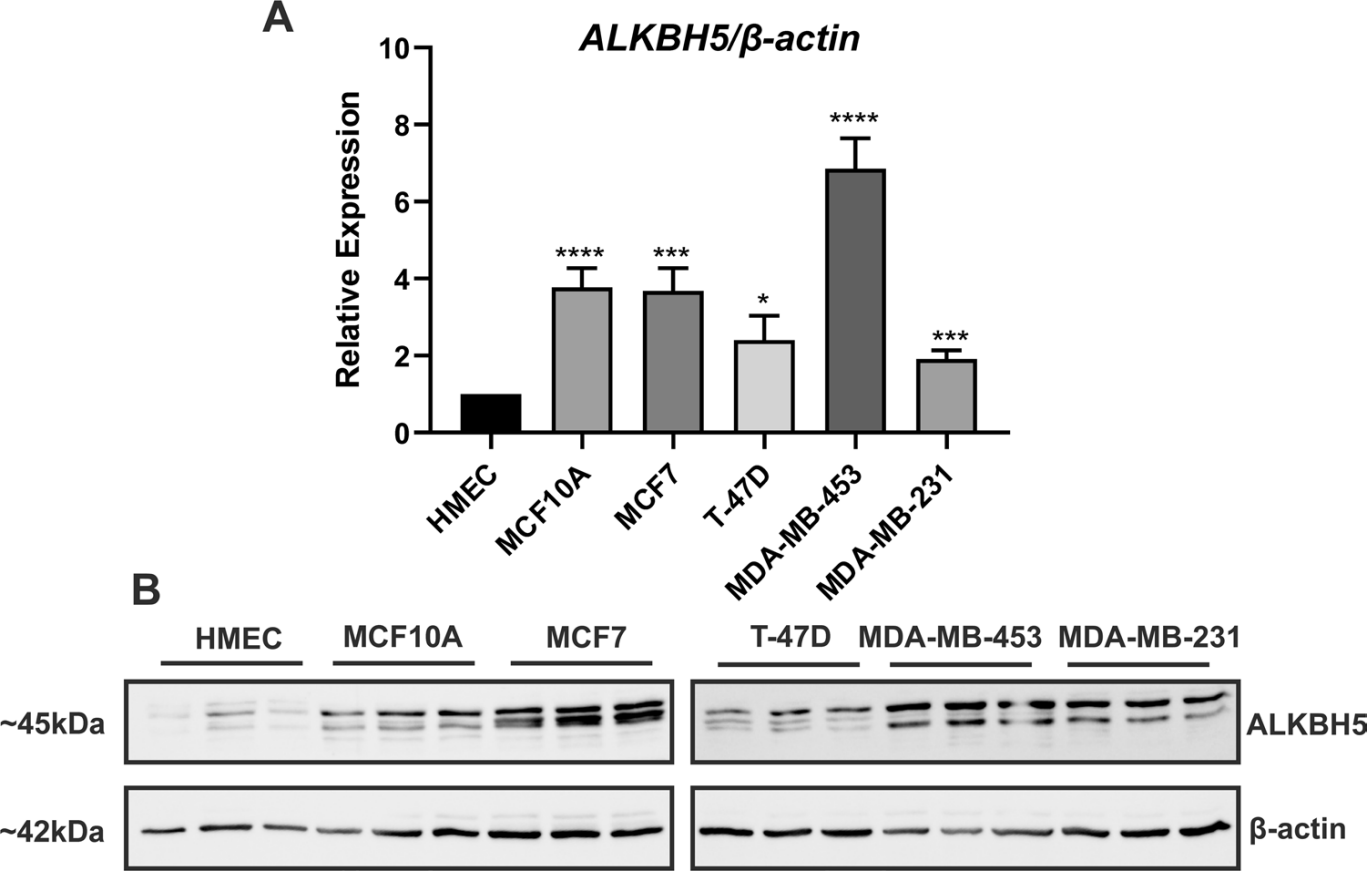
ALKBH5 basal expression in breast cell lines. **A** *ALKBH5* mRNA expression in primary human mammary epithelial cells (HMEC), non-malignant MCF10A and breast cancer MCF7, T-47D, MDA-MB-436, and MDA-MB-231 cell lines (n = 5/6). **B** Western blot showing ALKBH5 protein is expressed across the breast cell lines, β-actin was used as a loading control (n = 3). *P ≤ 0.05, ***P ≤ 0.001, ****P ≤ 0.0001 by t-test

**Fig. 2:**
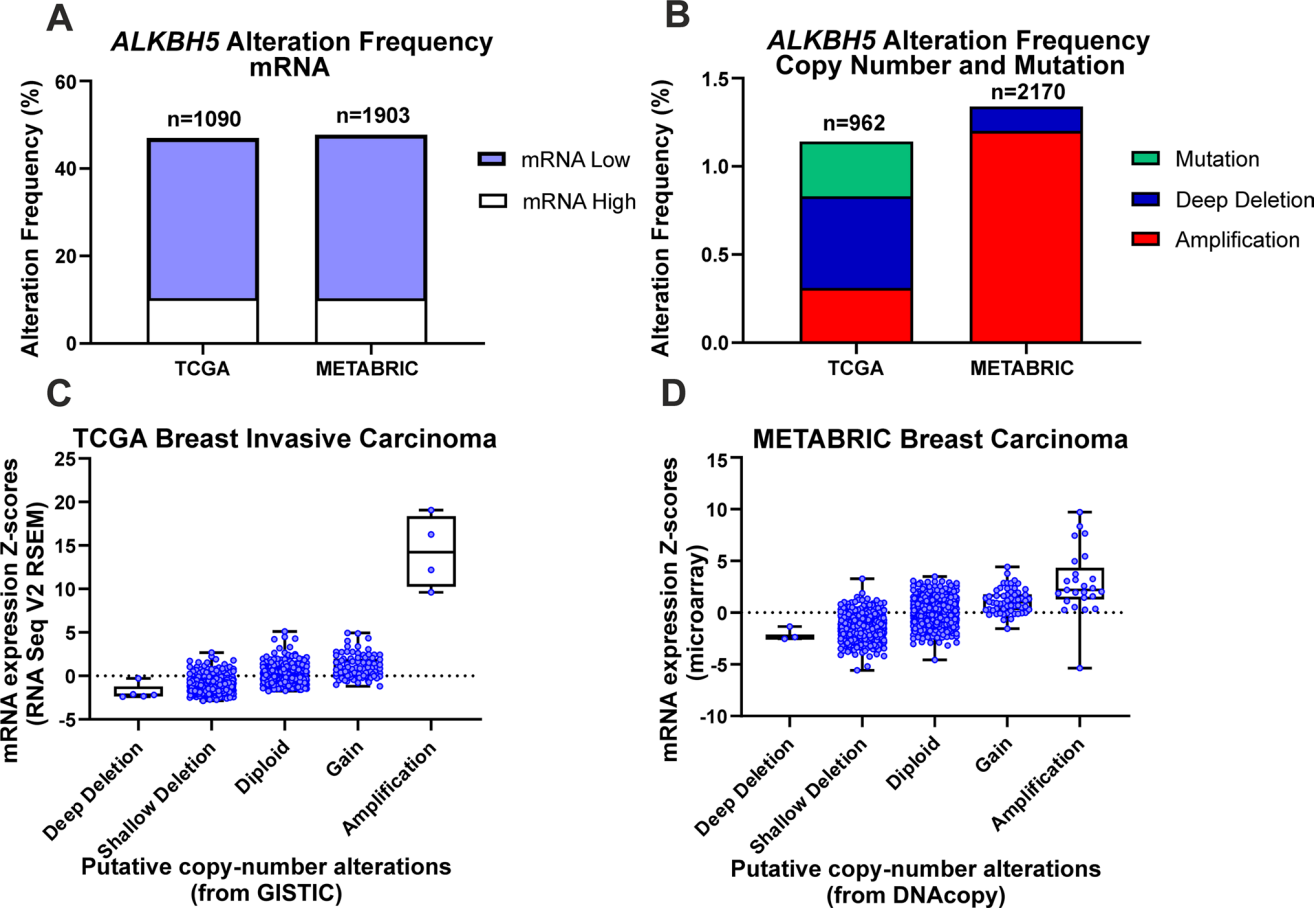
Bioinformatic analysis of *ALKBH5* in breast cancer datasets. The cBioPortal was used to investigate *ALKBH5* mRNA and copy number alterations (A-D) in breast cancer patients from the TCGA (Firehose Legacy) and METABRIC datasets

The basal expression of ALKBH5 was observed across breast cell lines at the mRNA and protein levels (Fig. 1A, B). In the TCGA dataset, 46.98% of samples showed *ALKBH5* mRNA expression alterations (Fig. 2A). Similarly, 47.71% of samples in the METABRIC cohort had *ALKBH5* mRNA alterations, the majority of which exhibited low mRNA expression, as compared with expression in the diploid samples (Fig. 2A). The TCGA dataset showed that 1.14% of patients harboured a copy number alteration (CNA), with 0.31% being amplification, 0.52% being deep deletion, and 0.31% being mutation (Fig. 2B). In the METABRIC cohort, 1.34% of patients had a CNA, with 1.2% being amplification, and 0.14% being deep deletion (Fig. 2B). Copy number gain was associated with high *ALKBH5* mRNA expression (p < 0.001; Fig. 2C, D and Supplementary Table 2). *ALKBH5* expression was lower in metastatic samples as compared to normal tissues (p < 0.05; Supplementary Fig. 2A).

**Fig. 3:**
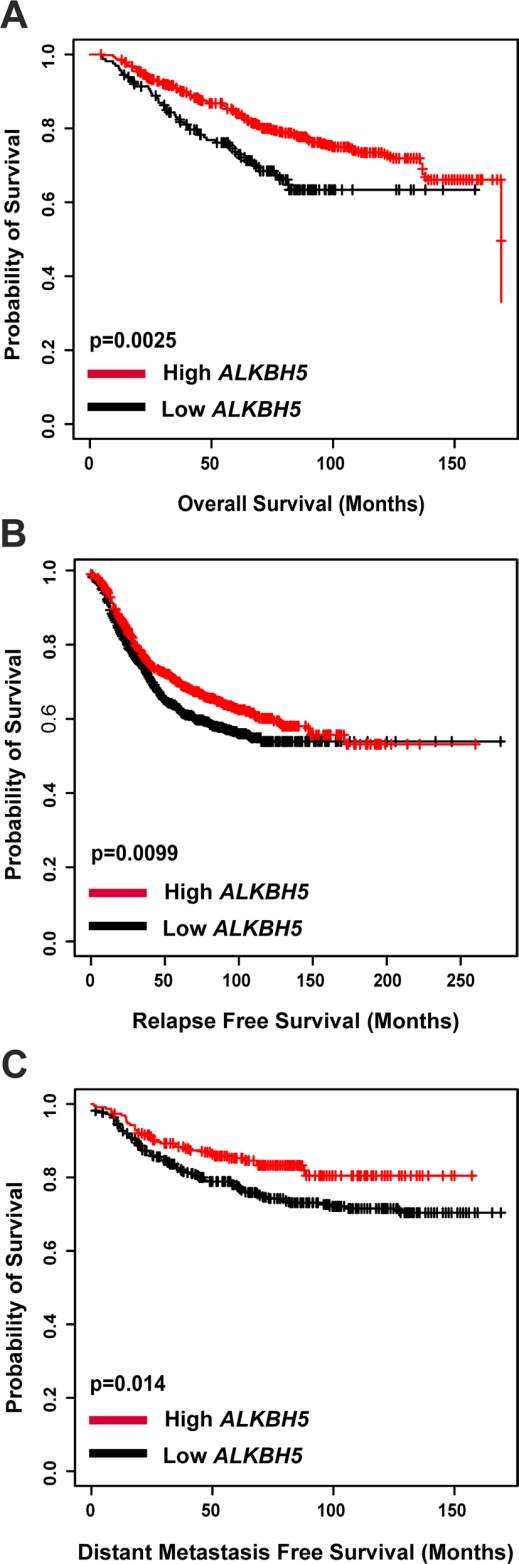
Kaplan–Meier plots was used to investigate *ALKBH5* mRNA expression and (**A**) overall survival (n = 626), (**B**) relapse free survival (n = 1764), and (**C**) distant metastasis free survival (n = 664)

The expression of *ALKBH5* was then correlated with survival. Low expression of *ALKBH5* was associated with shorter OS, RFS, and DMFS (p < 0.05; Fig. 3A–C). In the METABRIC dataset, low *ALKBH5* mRNA expression was significantly associated with factors pertinent to poor prognosis including larger tumour size, high grade, and higher NPI (p < 0.05; Supplementary Table 3). It was also observed that low *ALKBH5* expression was associated with shorter BCSS (p = 0.029; Supplementary Fig. 2B). Similar results were obtained utilising the bc-GenExMiner (Supplementary Table 4).

**Fig. 4:**
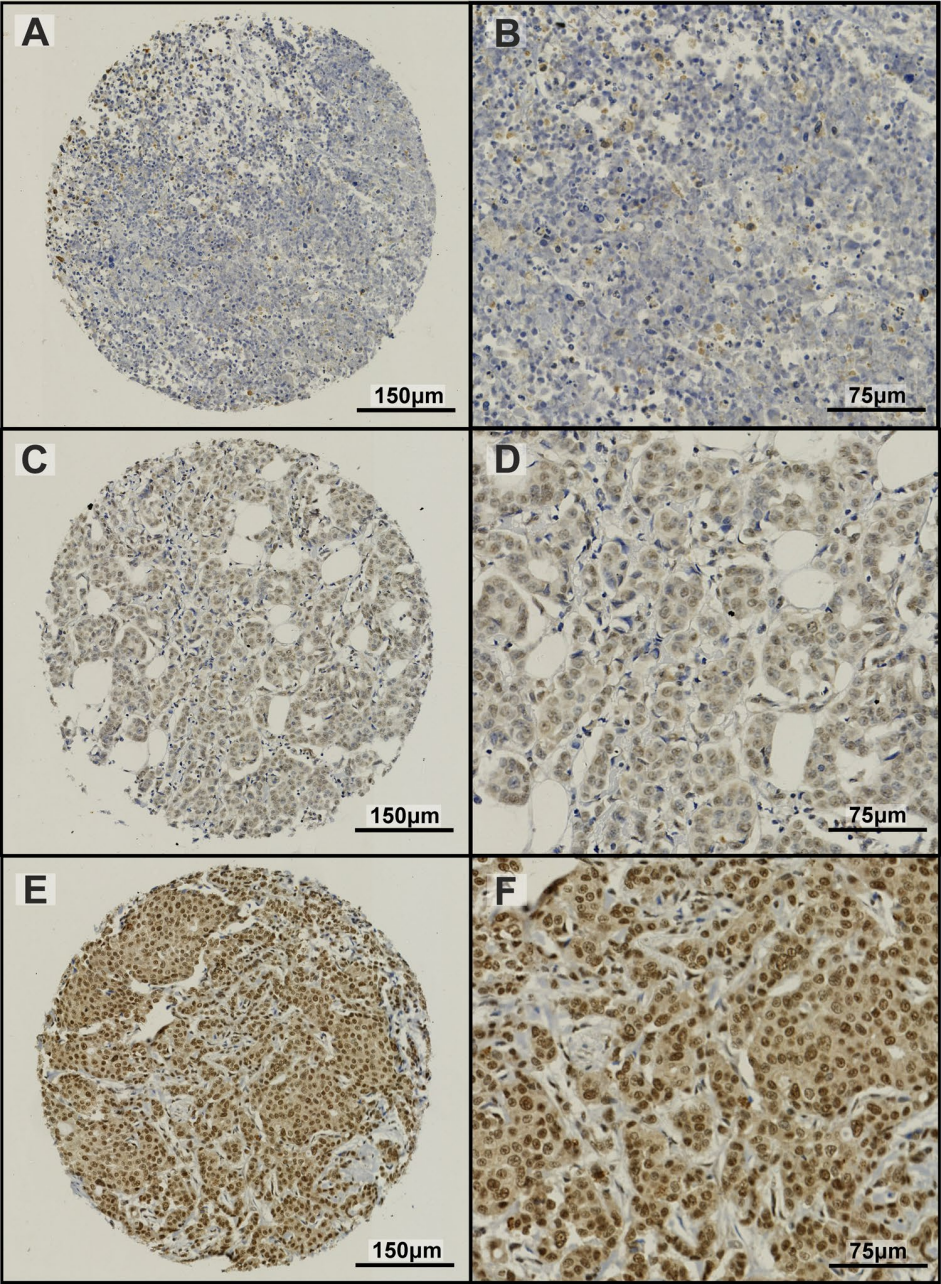
ALKBH5 immunohistochemical staining in the Nottingham Invasive BC TMA. A range of staining in the nuclear and cytoplasmic compartments was observed (**A**–**F**). Examples of weakly stained (**A**, **B**), moderately stained (**C**, **D**), and strongly stained (**E**, **F**) tumour samples are shown

**Fig. 5:**
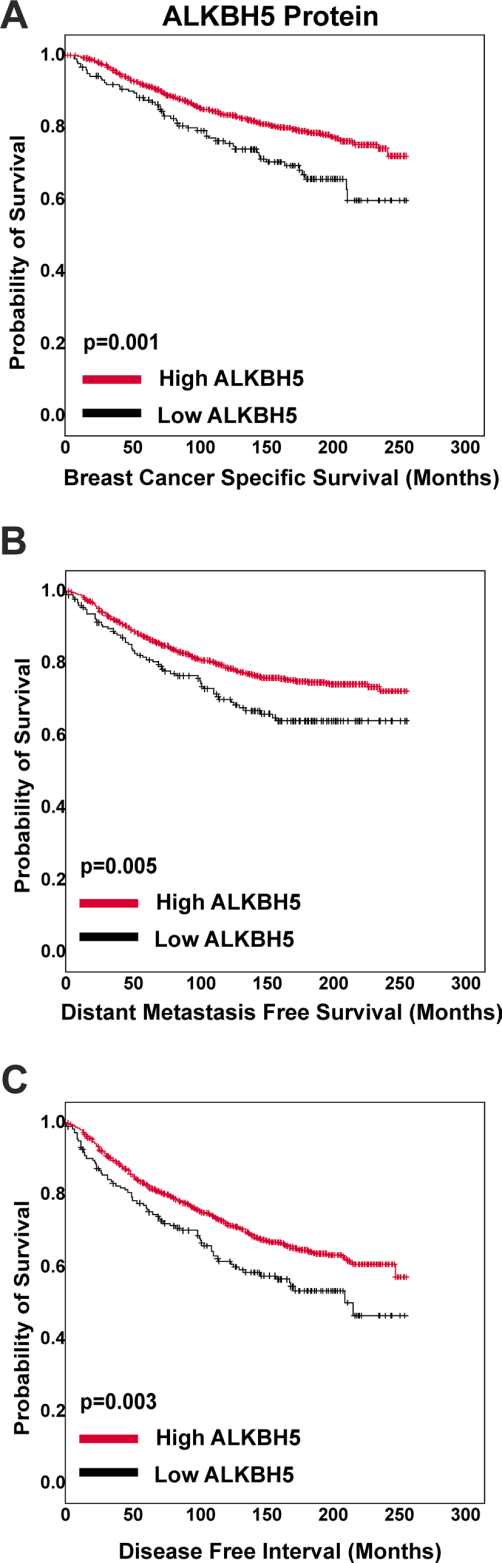
Kaplan–Meier plots was used to investigate ALKBH5 protein expression and (**A**) breast cancer specific survival, (**B**) distant metastasis free survival, and (**C**) disease free interval (n = 1318)

**Fig. 6:**
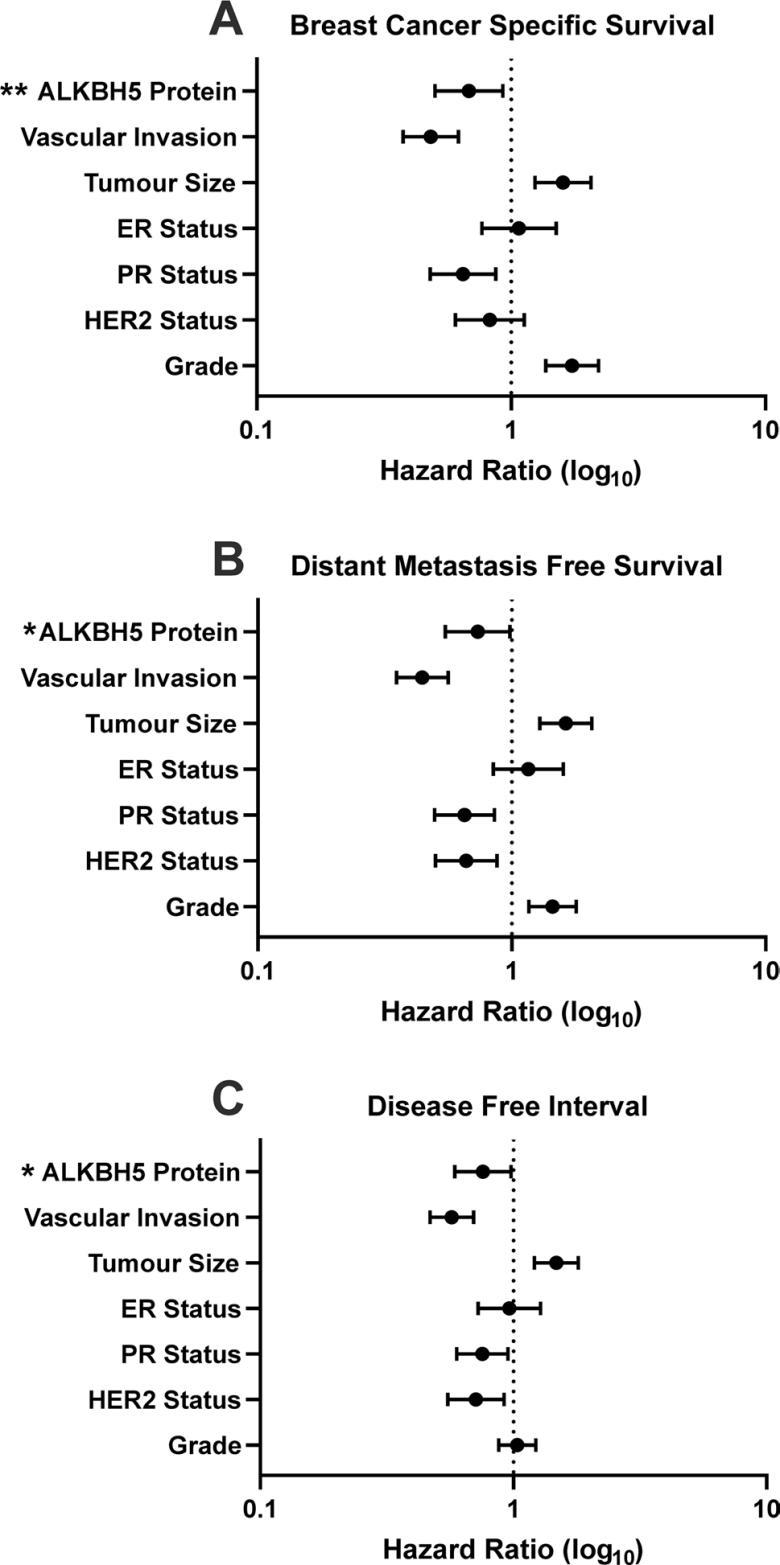
Forest plots showing the hazard ratios and 95% confidence interval of the multivariate survival analyses for ALKBH5 protein expression in the patient cohort for (**A**) breast cancer specific survival, (**B**) distant metastasis free survival, and (**C**) disease free interval. ALKBH5 protein expression was an independent prognostic factor

ALKBH5 protein expression in BC patient samples showed a range of staining in both the nuclei and cytoplasm of invasive tumour cells (Fig. 4). Supplementary Table 5 shows the association between ALKBH5 nuclear and cytoplasmic protein expression separately with clinicopathological parameters. Low ALKBH5 protein expression (combined nuclear and cytoplasmic expression) was significantly associated with a number of clinical parameters including larger tumour size, higher nodal stage, less tubule formation, presence of vascular invasion, hormone receptor negativity, and worse NPI prognostic group (p < 0.05, Table 1). Furthermore, low ALKBH5 protein expression was associated with worse prognosis (Fig. 5) and was a significant prognostic indicator, independent of other clinical parameters (Fig. 6, Supplementary Fig. 4 and Supplementary Table 6).

**Table 1:** Clinical associations with ALKBH5 combined protein expression in the Nottingham invasive breast carcinoma series

Parameters	ALKBH5 protein Expression		
	Low (%)	High (%)	p-value
Age			
< 50 years	52 (12.5)	364 (87.5)	0.609
≥ 50 years	122 (13.5)	780 (86.5)	
Tumour size			
< 2 cm	90 (11.6)	688 (88.4)	**0.035**
≥ 2 cm	84 (15.6)	456 (84.4)	
Grade			
1	18 (10.2)	158 (89.8)	**0.04**
2	59 (11.3)	465 (88.7)	
3	97 (15.7)	521 (84.3)	
Stage			
1	91 (11.5)	703 (88.5)	**0.012**
2	53 (14.1)	323 (85.9)	
3	30 (20.3)	118 (79.7)	
Tubule formation			
1	7 (10.1)	62 (89.9)	** < 0.001**
2	32 (8.2)	359 (91.8)	
3	135 (15.7)	723 (84.3)	
Pleomorphism			
1	0 (0)	15 (100)	0.315
2	48 (13.4)	311 (86.6)	
3	126 (13.3)	818 (86.7)	
Mitosis			
1	69 (11.5)	533 (88.5)	0.194
2	37 (13.7)	233 (86.3)	
3	68 (15.2)	378 (84.8)	
Multifocality			
No	124 (12.4)	876 (87.6)	0.066
Yes	50 (15.7)	268 (84.3)	
Tumour type			
NST	117 (13.2)	767 (86.8)	**0.016**
ILC, including lobular mixed	20 (19.2)	84 (80.8)	
Mixed NST and Iobular	12 (14.8)	69 (85.2)	
Mixed NST and special type	4 (10.5)	34 (89.5)	
Other Special tumour type including Mucinous, papillary, micropapillary, cribriform and adenoidcystic carcinoma	1 (11.1)	8 (88.9)	
Metaplastic carcinoma	2 (66.7)	1 (33.3)	
Tubular and tubular mixed	18 (9)	181 (91)	
Vascular invasion			
Negative	110 (12)	806 (88)	**0.033**
Positive	64 (15.9)	338 (84.1)	
Associated DCIS			
Negative	22 (10.9)	179 (89.1)	0.173
Positive	152 (13.7)	960 (86.3)	
LCIS			
Negative	148 (13.1)	983 (86.9)	0.252
Positive	26 (14.3)	156 (85.7)	
Lymph node status			
Negative	91 (11.5)	703 (88.5)	**0.015**
Positive	83 (15.8)	441 (84.2)	
ER			
Negative	55 (20.1)	219 (79.9)	** < 0.001**
Positive	118 (11.3)	925 (88.7)	
PgR			
Negative	91 (17)	444 (83)	** < 0.001**
Positive	79 (10.3)	691 (89.7)	
HER2			
Negative	144 (12.7)	990 (87.3)	0.12
Positive	28 (15.5)	153 (84.5)	
Triple negative			
No	135 (12.1)	978 (87.9)	**0.009**
Yes	36 (19)	153 (81)	
Ki67 index groups			
< 15 Hscore	59 (12.1)	430 (87.9)	0.356
≥ 15 Hscore	69 (14.1)	422 (85.9)	
Molecular classes			
Luminal types combined	118 (11.3)	925 (88.7)	** < 0.001**
HER2 enriched	17 (24.3)	53 (75.7)	
TNBC	36 (19)	153 (81)	
Nottingham Prognostic Index			
Good prognostic group	41 (9.8)	378 (90.2)	**0.006**
Moderate prognostic group	92 (13.6)	582 (86.4)	
Poor prognostic group	41 (18.2)	184 (81.8)	
Menopausal status			
Pre	56 (12)	410 (88)	0.135
Post	118 (13.8)	734 (86.2)	
Chemotherapy			
Non treated	92 (11)	745 (89)	**0.004**
Treated	82 (17.1)	398 (82.9)	
Endocrine therapy			
Non treated	70 (16.4)	357 (83.6)	**0.013**
Treated	104 (11.7)	787 (88.3)	
Radiotherapy local			
Non treated	50 (13.4)	322 (86.6)	0.206
Treated	124 (13.1)	822 (86.9)	
Radiotherapy LNs			
Non treated	123 (12.1)	894 (87.9)	**0.02**
Treated	51 (16.9)	250 (83.1)	
Biological therapy			
Non treated	130 (13)	873 (87)	0.34
Treated	10 (14.3)	60 (85.7)	

Statistically significant associations are highlighted in bold

**Fig. 7:**
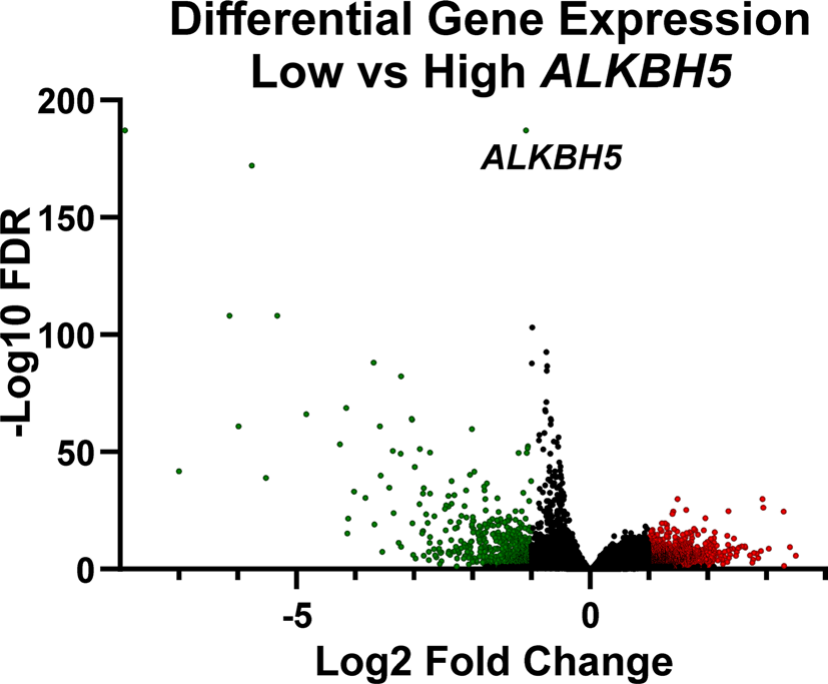
The TCGA RNA-seq dataset was stratified into low and high *ALKBH5* expression by quartile, and the differentially expressed genes analysed using DeSeq2. Genes with significantly higher expression in low *ALKBH5* are coloured red and genes significantly lower in low *ALKBH5* are coloured green. Non-significantly differentially expressed genes are plotted in black. Significant gene expression: FC ± 2 and FDR < 0.05

Given the evidence suggesting that low ALKBH5 expression is associated with worse outcome, the TCGA-BRCA cohort was stratified into low and high *ALKBH5* expression, and DEGs identified. A total of 1964 DEGs were identified, 594 with lower expression, and 1321 more highly expressed in samples with low as compared to high *ALKBH5* expression (Fig. 7 and Supplementary Table 7). Genes higher in tumours with low *ALKBH5* expression were significantly enriched (FDR < 0.05) in the cytokine-cytokine receptor interaction pathway (Supplementary Table 8). In the lower DEGs in tumours with low *ALKBH5* expression, 14 KEGG pathways were significantly enriched (FDR < 0.05). This included neural related pathways such as neuroactive ligand-receptor interaction, glutamatergic synapse, and dopaminergic synapse pathways (Supplementary Table 8).

## Discussion

Whilst alterations in gene expression in BC have been extensively studied [49, 50], the contribution of covalent mRNA modifications such as m^6^A is still largely unknown. ALKBH5 is a m^6^A RNA demethylase [6], and the role of m^6^A components in cancer is only just being revealed. Several recent studies reported that reduced ALKBH5 expression in BC cell lines decreased viability, migration, invasion and tumour growth and metastasis in mouse models [29, 31, 32]. Mechanistically, hypoxia dependant expression of ALKBH5 promotes a BC stem cell phenotype [28, 29]. Despite the fundamental importance of m^6^A in cancer, the exact clinical relevance of ALKBH5 remains elusive as ALKBH5 protein and mRNA expression have been shown to be both increased [23, 31] or decreased [22, 31] in tumours compared to normal tissue. High *ALKBH5* mRNA expression has previously been associated with ER-positive and PR-positive patients [31], suggesting that ALKBH5 may have distinct clinical significance depending on BC subtype. To address this, our study assessed the clinical association of ALKBH5 in a large well characterised cohort of BC patients.

ALKBH5 mRNA and protein expression was confirmed in malignant and non-malignant breast cell lines. Higher *ALKBH5* mRNA expression was observed in transformed (MCF10A) and malignant cells compared to non-malignant primary HMEC at the mRNA level. ALKBH5 expression was increased, and m^6^A decreased, after immortalisation and oncogenic transformation of primary HMEC cells [30, 31], supporting a role for ALKBH5 in the progression and transformation of cells from a non-malignant to a malignant state.

Recent investigations into the prognostic value of *ALKBH5* mRNA expression showed no clear association [23, 31, 51]. However, a recent study showed high *ALKBH5* to be a predictor of poor survival in triple-negative BC (TNBC) patients [27]. At the mRNA level, the KM-Plotter, METABRIC dataset, and bc-GenExMiner revealed that low *ALKBH5* was associated with unfavourable outcome. Additionally, the expression of *ALKBH5* was found to be lower in metastatic samples compared to normal tissues, suggesting that lower *ALKBH5* expression could lead to an increased chance of developing metastasis. Bioinformatic analysis using the cBioPortal for Cancer Genomics revealed that a small number of patients had CNA or mutation of *ALKBH5* in both BC datasets investigated. In contrast to these modest changes in copy number, a large number of changes in *ALKBH5* mRNA expression was observed with the majority of these resulting in lower mRNA expression. However, given the limited number of copy loss or loss of function mutations identified to date in BC patients, it is likely other mechanisms, such as epigenetic down-regulation of expression may also play a role in reduced *ALKBH5* expression in BC.

Given the results on the mRNA level, ALKBH5 protein expression was assessed in a large cohort of well characterised BC patient samples. Consistent with previous studies in cancer, a range of ALKBH5 staining was identified in the nuclear and cytoplasmic compartments of cells [16, 23, 29, 52, 53]. Analysis of ALKBH5 nuclear and cytoplasmic staining separately revealed few associations with the clinicopathological parameters. However, low ALKBH5 combined protein expression was associated with parameters of poor prognosis in BC and worse survival.

To explore the functional role of ALKBH5 in BC, the TCGA BC primary tumour RNA-sequencing dataset was used to identify DEGs with low and high *ALKBH5* expression. This revealed 1964 DEGS, 594 down-regulated, and 1321 DEGs up-regulated when *ALKBH5* expression is lower. KEGG pathway revealed up-regulated DEGs were enriched in the cytokine-cytokine receptor interactions pathway. Cytokines in the tumour microenvironment play an important role in tumour pathogenesis, including in promoting metastasis [54]. Immune cells are attracted by oncogenic changes, and these cells secrete cytokines, chemokines, and growth factors to which the tumour responds leading to tumour development and progression [55]. Thus, suggesting tumours with low *ALKBH5* have an increased cytokine signalling causing pro-survival and pro-metastatic signals. In addition, several studies have investigated the role of m^6^A RNA methylation and the immune system. The METTL3 m^6^A methyltransferase is important for T cell homeostasis and differentiation [56], and dendritic cell maturation and activation [57]. Recent investigations have implicated m^6^A in response to immunotherapy, an emerging and increasingly used therapy now being utilised in BC. Regulators of m^6^A influence the tumour immune microenvironment and response to anti-PD1 therapies [58–62]. Two matrix metalloproteinases (MMPs; MMP-1 and MMP-20) were identified as significantly up-regulated in tumours with low *ALKBH5*. MMPs play a pivotal role in cancer cell migration, invasion, and metastasis [63]. Multiple studies show MMP-1 plays a role in invasiveness by promoting local growth and the formation of metastasis [64–66]. Despite initial reports that MMP-20 expression was restricted to enamel, it is expressed in BC cell lines and tissue and promotes invasion in ovarian cancer [67, 68].

Fourteen significantly enriched pathways with down-regulated DEGs in tumours with low *ALKBH5* expression were identified. The majority were related to neural signalling, including neuroactive ligand-receptor interactions, glutamatergic synapse, and dopaminergic synapse. Neuroactive ligand-receptor interaction was the most enriched pathway which has been shown to play a role in brain metastasis in TNBC [69]. Dopamine functions in many pathways through binding to its receptor. There is currently conflicting evidence on the role of dopamine receptor activation in cancers, including in BC [70, 71], however several studies have shown that stimulated dopamine signalling inhibits tumour growth [72–74]. Dopamine receptor D2 (*DRD2*) was down-regulated with low *ALKBH5,* and studies have shown DRD2 to be up or down-regulated in different cancer types [75–77]. A study has also implicated FTO in the control of DRD2 dependant signalling [78].

Altered metabolism is a widely accepted hallmark of cancer [79], and increased glutamine and glutamate signalling, including through up-regulation of receptors, increases cancer cell growth and proliferation [80]. Enrichment of this pathway suggests that glutamate signalling is down-regulated in these tumours. Whilst many studies have associated the increase of these receptors to be oncogenic, previous research has found that in cancer cells the inhibition of certain glutamate receptor subunits has led to the increased proliferation [81, 82]. Interestingly, glutamate metabotropic receptor 4 (GRM4) was down-regulated with low *ALKBH5* expression. In BC high expression of GRM4 was associated with better prognosis in patients and furthermore may act as a tumour suppressor [83].

While this study presents promising findings of the potential role of ALKBH5 in invasive BC at both the mRNA and protein level using multiple large well-characterised cohorts. However, we acknowledge some limitations. Firstly, the protein expression BC cohort used in this study is a retrospective cohort. While these results were validated on additional publicly available BC transcriptomic cohorts, extending this study to include further patient cohorts, including ethnically diverse patient cohorts to further understand the prognostic value of ALKBH5 would be beneficial. In addition, in vitro and in vivo studies would allow for further insights to be gained into the mechanistic role of ALKBH5 in BC and provide a wider mechanistic context to its clinical relevance. Additionally, utilising a range of cell line and patient derived models representing different molecular subtypes of BC would allow for further characterisation of the role of ALKBH5 in BC.

Taken together, this study provides clinical evidence that ALKBH5 plays a role in BC. Low ALKBH5 mRNA and protein expression was shown to be associated with unfavourable clinical outcomes and worse prognosis. Further functional studies into the role of ALKBH5 and related mechanisms are therefore warranted to determine how reduced expression of ALKBH5 may contribute to poorer outcomes in BC. Given ALKBH5 functions as an m^6^A demethylase, the association of low ALKBH5 with poorer outcomes indicates that unopposed RNA m^6^A methylation mediated by METTL3 may promote BC progression. For this reason, a phase 1 clinical trial of the STC-15 METTL3 inhibitor (NCT05584111) is currently underway [84], and extending this to BC is justified [85].

### Supplementary Information

Supplementary Material 1.

Supplementary Material 2.

## Data Availability

The transcriptomic and associated clinical data utilised in this study is publicly available from the cBioPortal for Cancer Genomics [38, 39], KM-Plotter [43], bc-GenExMiner v4.7 database [44], and the UCSC Xena browser [45]. The authors confirm the data is available on reasonable request.
