# Supplementary material for: The role of the ALKBH5 RNA demethylase in invasive breast cancer
Source: Discov Oncol. 2024 Aug 11;15:343. doi: 10.1007/s12672-024-01205-8 (PMC11317455; doi:10.1007/s12672-024-01205-8)
Supplement: Supplementary file 1 — Supplementary Material 1. [file 12672_2024_1205_MOESM1_ESM.docx]

**The role of the ALKBH5 RNA demethylase in invasive breast cancer**

**

**

**Supplementary Fig. 1**

Full, uncropped western blot images showing basal expression of ALKBH5 (A) with β-actin (B) as a loading control from Fig 1B.


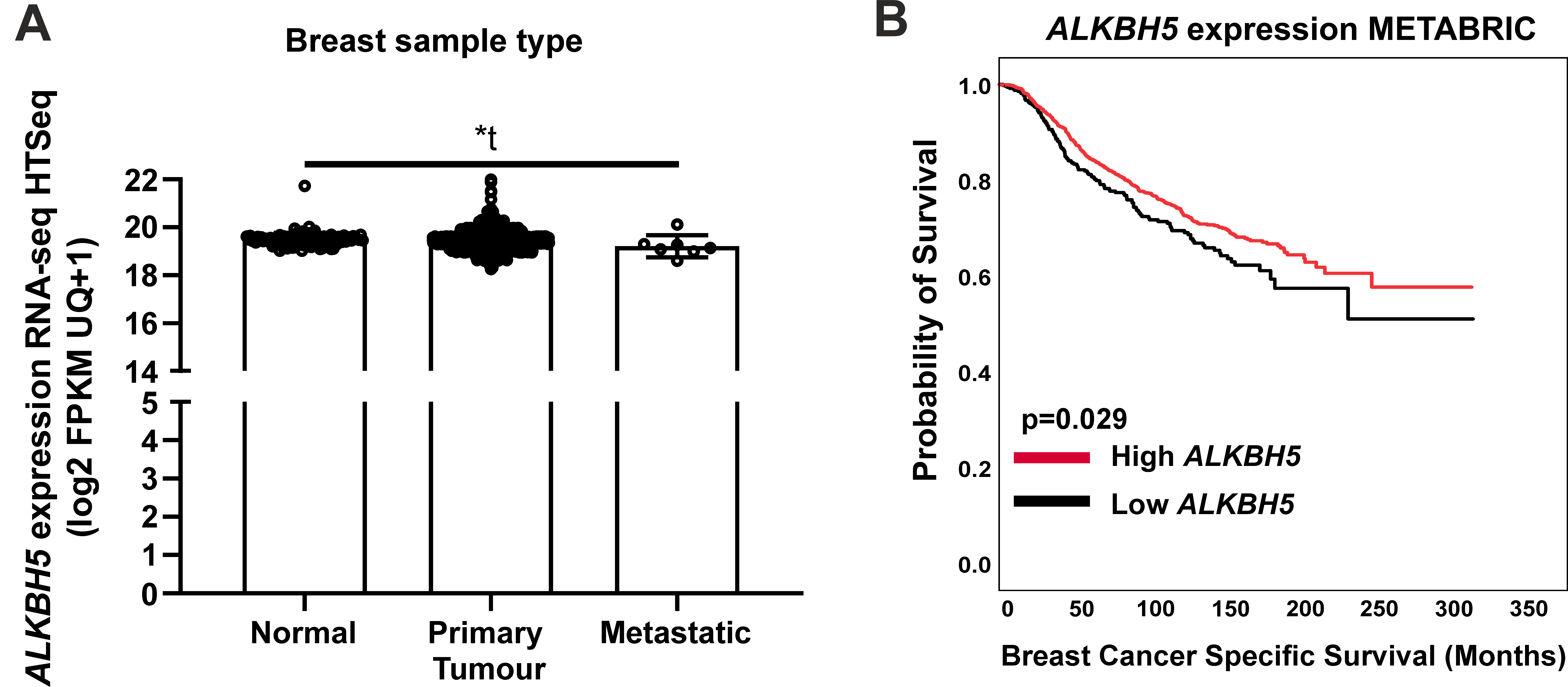


**Supplementary Fig. 2**

*ALKBH5* mRNA expression in breast sample type and outcome. (A) The GDC TCGA BC dataset was used to assess *ALKBH5* mRNA expression in normal (n=113), primary (n=1097), and metastatic breast samples (n=7), * = p ≤ 0.05. (B) *ALKBH5* expression in the METABRIC dataset was dichotomised into low and high expression and Kaplan-Meier survival analysis used to determine prognostic significance (n=1977).





**Supplementary Fig. 3**

Kaplan–Meier survival plots were used to investigate the association of ALKBH5 nuclear (A-C) and cytoplasmic (D-F) protein expression with breast cancer specific survival (A and D), distant metastasis free survival (B and E), and disease free interval (C and F) (n=1318).


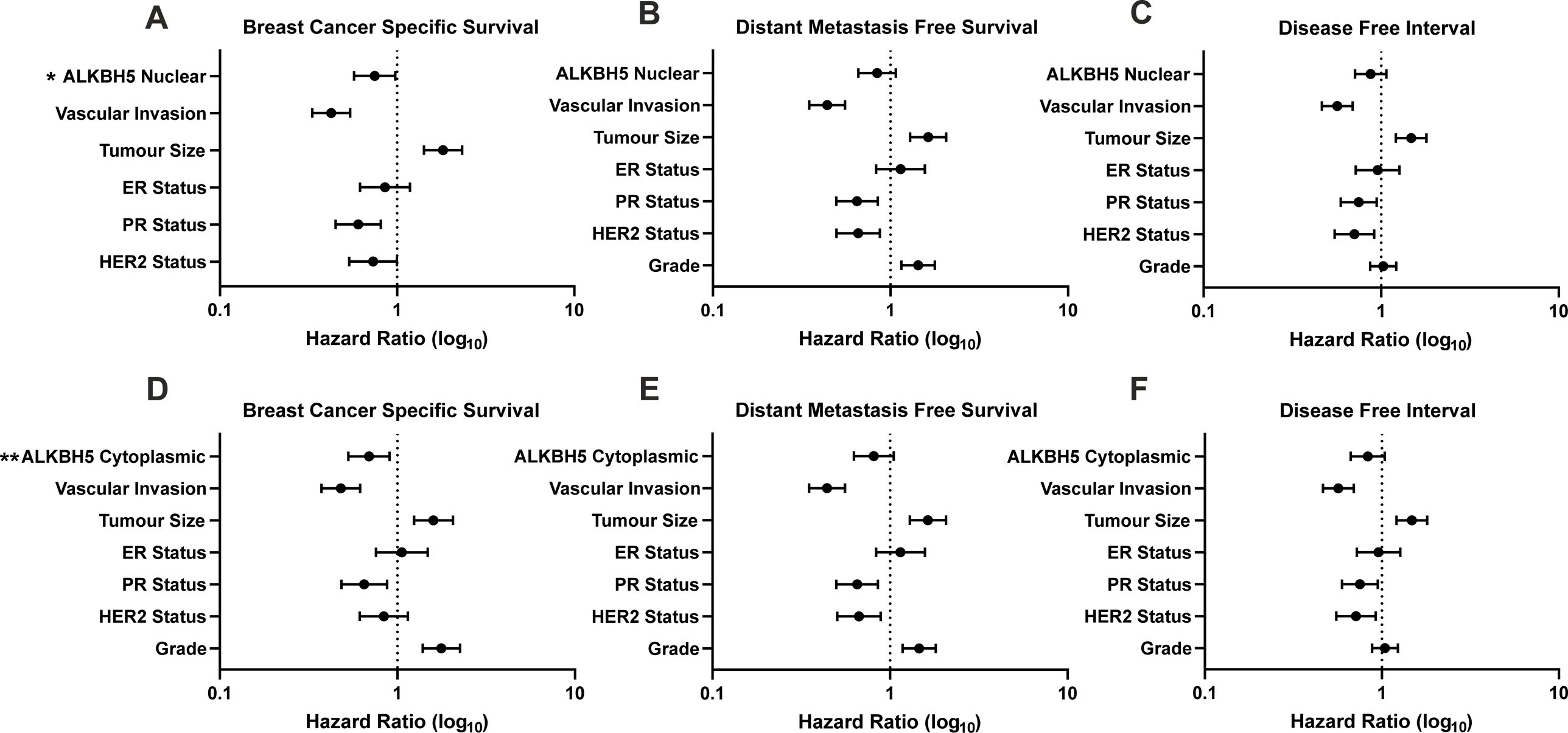


**Supplementary Fig. 4**

Forest plots showing the hazard ratios and 95% confidence interval of the multivariate survival analyses for ALKBH5 nuclear (A-C) and cytoplasmic (D-F) protein expression in the patient cohort for (A and D) breast cancer specific survival, (B and E) distant metastasis free survival, and (C and F) disease free interval.
